# Prevalence of Chronic Kidney Disease in a High-Risk Population in Urban Lahore, Pakistan: A Cross-sectional Study

**DOI:** 10.7759/cureus.63296

**Published:** 2024-06-27

**Authors:** Afifa Khan, Manal F Cheema, Rida Fatima, Sidra S Cheema, Zeeshan Butt, Samreen Gillani, Ayesha Ahmad, Muhammad Subhan Ullah, Urba Jalal, Shafiq Cheema

**Affiliations:** 1 Department of Nephrology, Jinnah Hospital, Lahore, PAK; 2 College of Medicine, CMH (Combined Military Hospital) Lahore Medical College and Institute of Dentistry, Lahore, PAK; 3 Department of Internal Medicine, Jinnah Hospital, Lahore, PAK; 4 Department of Pathology, CMH (Combined Military Hospital) Lahore Medical College and Institute of Dentistry, Lahore, PAK; 5 Department of Internal Medicine, Baystate Medical Center, Springfield, USA; 6 Department of Internal Medicine, Fatima Memorial Hospital, Lahore, PAK

**Keywords:** ckd in pakistani population, hypertensive ckd, diabetic kidney disease (dkd), chronic kidney disease prevalence, chronic kidney disease factors, chronic kidney disease (ckd)

## Abstract

Background

Chronic kidney disease (CKD) is a globally increasing health concern, and there is a growing focus on early screening and prevention efforts. However, the availability of data on CKD prevalence in Pakistan, particularly in the urban area of Lahore district, is limited. The objective of the Kidney Early Evaluation Program (KEEP) Lahore was to assess the prevalence of CKD in a high-risk population residing in the urban area of Lahore, Pakistan.

Methods

A cross-sectional study was conducted involving 254 participants, who were over 18 years old and belonged to a high-risk population according to the pre-defined operational definitions. The participants were randomly selected from various towns in Lahore. Screening camps were set up to measure serum creatinine levels and urinary albumin to creatinine ratio (UACR), and then the estimated glomerular filtration rate (eGFR) was calculated using the CKD Epidemiology Collaboration 2021 (CKD EPI) equation.

Results

Out of the total 254 participants, a diagnosis of CKD was made in 62 (24.2%) individuals. Significant associations were found between CKD and risk factors including hypertension, diabetes, family history of CKD, ischemic heart disease (IHD) or congestive heart failure (CHF), intake of painkillers, and herbal medicines. However, no association was found between obesity (BMI ≥ 30) and CKD. Participants diagnosed with CKD had a mean age of 49.9 years and a mean serum creatinine level of 1.2 mg/dL, while non-CKD participants had a mean age of 43.7 years and a mean serum creatinine level of 0.7 mg/dL.

Conclusion

Our study revealed that CKD was prevalent in about one-fourth of the participants from the high-risk population of Lahore, indicating a high prevalence of the disease within society. Moreover, hypertension, diabetes, family history of CKD, heart disease, painkillers, and the use of herbal medicines were all significantly linked to CKD in the surveyed sample population.

## Introduction

Chronic kidney disease (CKD) is a significant contributor to morbidity and mortality related to non-communicable diseases, making it a crucial public health concern worldwide [1]. A recent meta-analysis reported global prevalence of CKD stages 1-5 at 843.6 million or 13.4% [2]. In earlier studies, the global prevalence of CKD is estimated to be 9.1%, and it is associated with approximately 1.2 million deaths annually [3]. However, these reported cases represent only a fraction of the actual disease burden, as a substantial number of cases remain undiagnosed. As many as nine out of 10 individuals with CKD in resource-poor settings with weak primary care infrastructure are unaware that they have this condition and therefore do not seek treatment [4].

The prevalence of CKD varies across different regions of the world, with developing countries experiencing particularly high rates [5]. The CKD prevalence in Southeast Asia ranges from 7.0% to 34.3% [6]. In Pakistan, studies have reported a prevalence of CKD ranging from 12.5% to 29.9% [7-9]. Additionally, CKD is strongly associated with advanced age, diabetes, hypertension, and ischemic heart disease (IHD), suggesting a higher prevalence within these high-risk patient groups [9]. For instance, a survey conducted on high-risk patients attending community health clinics reported a CKD prevalence of 68.9% among these individuals [10]. Nevertheless, there is a lack of comprehensive local studies that specifically determine the disease burden among high-risk patients in urban Lahore, Pakistan.

The objective of our study, Kidney Early Evaluation Program (KEEP) Lahore, was to assess the prevalence of CKD in the high-risk population of Lahore city. Literature suggests that the prevalence of CKD can vary depending on the prevalence of associated risk factors and the method used to determine the estimated glomerular filtration rate (eGFR). Therefore, in the absence of local data, this study aims to provide valuable insights into the prevalence of CKD among our high-risk population. It will help to establish an understanding of the local disease burden of CKD and guide the development of guidelines for early diagnosis and a multidisciplinary approach involving nephrologists and clinicians. This collaborative effort will focus on screening and managing complications to reduce the morbidity and mortality associated with CKD.

## Materials and methods

Study design

This cross-sectional study was conducted over one year, from January 1, 2022, to December 31, 2022, in randomly selected urban towns of Lahore. Lahore, the capital city of Punjab, Pakistan, is divided into several administrative districts and Union Councils. Six out of 10 administrative towns in Lahore were randomly chosen through simple random sampling. The number of high-risk cases from each town was determined using proportionate sampling based on the town's population. To ensure representation of the high-risk population, a multi-stage sampling technique was employed. From each town, union councils near secondary or tertiary healthcare centers were selected using convenience sampling.

Sample size

A sample size of 254 cases was determined for the urban population of Lahore, which has approximately 8.08 million people. This calculation was based on a 95% confidence level with a 1% margin of error, considering an expected CKD prevalence of 12.5%, the lowest among all possibilities.

Study criteria

The inclusion criteria were patients aged 18 and older, both male and female, and high-risk individuals (as per the operational definition below) attending screening camps in urban areas of Lahore. The exclusion criteria were patients with known CKD based on their history and medical records, those with a history of dialysis or renal transplant, and patients unwilling to participate in the study.

Ethical considerations

The study was approved by the Ethical Review Board Allama Iqbal Medical College/Jinnah Hospital, Lahore (approval number: 59th/ERB dated May 14, 2020). Data confidentiality was maintained through anonymization and secure storage. Informed consent was obtained from volunteers at the camps before including them in the study.

Procedure and assessments

Screening camps were set up in the main secondary and tertiary care health facilities in these towns. The high-risk population was invited to participate in the free screening through advertisements detailing the date, time, and location of the camps one week in advance. Demographic information was collected using a well-designed form. Participants were directed to the Chughtai Laboratory collection center for free sampling. Blood samples were taken under aseptic conditions to measure serum creatinine levels, with venipuncture performed by expert phlebotomists. Random or spot urine samples were collected to determine the urinary albumin to creatinine ratio. Laboratory results were used to calculate the eGFR, and the presence of CKD was identified according to the operational definition. Results were shared with the participants, and those diagnosed with CKD were referred to nephrologists for further evaluation. 

*Operational Definitions*

High-risk patients: Patients having any of the following conditions were labeled as high-risk: Hypertension (at least two resting blood pressure readings of ≥ 130/80 mmHg taken 20 minutes apart using standard protocol), diabetes mellitus (fasting blood sugar level ≥126 mg/dl or glycated hemoglobin (HbA1C) ≥ 6.5 mmol or taking antidiabetic treatment, age (above 60 years), family history of CKD among parents, siblings, or children, history of prior or current kidney stones, and history of hepatitis C virus (HCV) infection and IHD or cardiomyopathy.

CKD: Elevated random urine albumin to creatinine ratio (UACR) or low eGFR (UACR of ≥30 mg/g and or eGFR of ≤ 60ml/min/1.73m^2^) was labeled as CKD. Estimated GFR was calculated by CKD Epidemiology Collaboration 2021 (CKD EPI) creatinine equation [11].

Painkiller use: Daily intake of non-steroidal anti-inflammatory drugs (NSAIDs) for a total period of four weeks during the last three months.

Statistical analysis

We used IBM SPSS Statistics for Windows, Version 24.0 (Released 2016; IBM Corp., Armonk, New York, United States) for analyzing the results of our study. Numerical variables like age were summarized using mean and standard deviation. Qualitative variables like sex and CKD were presented as prevalence tables. Chi-square test was applied to check statistical significance post stratification based on patient characteristics and a p-value < 0.05 was taken as significant.

## Results

Baseline characteristics

The study examined a total of 254 patients. The mean age of the population was 42.26±13.66 years. Among them, 130 (51.2%) were females, while 124 (48.8%) were males. Hypertension was present in 51.6% of the study population, diabetes in 42.9%, and a family history of CKD in 58.3%. Furthermore, 79.1% of the participants had a body mass index (BMI) of less than 30. Out of the 254 patients, 62 (24.4%) were diagnosed with CKD. Table 1 provides details on the frequencies and percentages of the baseline characteristics of the study population.

**Table 1:** Baseline characteristics of patients included in the study (N=254) CKD: chronic kidney disease; IHD: ischemic heart disease; CHF: congestive heart failure; BMI: body mass index; eGFR: estimated glomerular filtration rate

Qualitative Characteristics		
Characteristic	Finding	Frequency (Percentage)
Gender	Male	124 (48.8%)
	Female	130 (51.2%)
Hypertension	Yes	131 (51.6%)
	No	123 (48.4%)
Diabetes	Yes	109 (42.9%)
	No	145 (57.1%)
Family history of CKD	Yes	149 (58.3%)
	No	105 (41.7%)
Hepatitis C infection	Yes	44 (17.3%)
	No	210 (82.7%)
IHD or CHF	Yes	47 (17.3%)
	No	207 (82.7%)
History of kidney stones	Yes	54 (21.3%)
	No	200 (78.7%)
Smoking	Yes	40 (15.7%)
	No	214 (84.3%)
Painkiller intake	Yes	49 (19.3%)
	No	205 (80.7%)
Herbal medicine intake	Yes	32 (12.6%)
	No	222 (87.4%)
Cancer history	Yes	2 (0.8%)
	No	252 (99.2%)
Obesity	BMI<30	201 (79.1%)
	BMI>30	53 (20.9%)
CKD status	Present	62 (24.4%)
	Absent	192 (75.6%)
Quantitative Characteristics		
Characteristic	Unit	Mean ± Standard Deviation
Age	Years	45.26 ± 13.66
Body Mass Index	Kg/m^2^	26.64 ± 4.47
Serum Creatinine	mg/dl	0.85 ± 0.44
Urine Albumin Creatinine Ratio	mg/g	252.08 ± 1141.42
eGFR	ml/min	73 ± 25

Comparative analysis

Hypertension, diabetes, family history of CKD, IHD, or congestive heart failure (CHF), use of painkillers, and herbal medicines intake were found to be significantly associated with CKD in the sample population surveyed. Out of 109 diabetic patients, 40 had CKD. Diabetic patients were found to have a 3.2 times greater risk of having CKD, while hypertensive patients had a 1.8 times greater risk of having CKD. Thirty-nine patients out of a total of 131 with hypertension were found to have CKD. Another risk factor strongly associated with CKD was the presence of IHD or CHF, which if present increased the risk of developing CKD by 2.9 times. Out of 49 patients who had a history of painkiller intake, 20 were found to have CKD. Similarly, out of 32 patients with herbal medicine intake, 15 developed CKD. Both factors were found to be strongly associated with the development of CKD. Similarly, having a family history of CKD was associated with 2.84 times the risk of having CKD in the studied population (P<0.05). No significant association was found between CKD and patients who were obese (BMI≥30) or had a history of smoking, HCV, cancer, or kidney stones. Table 2 shows the association of various risk factors with the prevalence of CKD.

**Table 2:** Association of various categorical risk factors with CKD CKD: chronic kidney disease; IHD: ischemic heart disease; CHF: congestive heart failure; BMI: body mass index

Variable			CKD		Odds Ratio	95% Confidence interval	P-value
			Yes	No			
Gender	Male	n/N (%)	28/124 (22.5%)	96/124 (77.5%)	1.214	0.684-2.157	0.508
	Female	n/N (%)	34/130 (26.1%)	96/130 (73.9%)			
Hypertension	Yes	n/N (%)	39/131 (29.7%)	92/131 (70.3%)	1.843	1.024-3.318	0.040
	No	n/N (%)	23/123 (18.6%)	100/123 (81.4%)			
Diabetes	Yes	n/N (%)	40/109 (36.7%)	69/109 (63.3%)	3.241	1.782-5.894	0.000
	No	n/N (%)	22/145 (15.1%)	123/145 (84.9%)			
Family history of CKD	Yes	n/N (%)	46/149 (30.9%)	103/149 (69.1%)	2.484	1.316-4.691	0.004
	No	n/N (%)	16/105 (15.2%)	89/105 (84.8%)			
Hepatitis C infection	Yes	n/N (%)	7/44 (15.9%)	37/44 (84.1%)	0.533	0.225-1.266	0.149
	No	n/N (%)	55/210 (26.2%)	155/210 (73.8%)			
IHD/CHF	Yes	n/N (%)	20/47 (42.5%)	27/47 (57.5%)	2.910	1.489-5.688	0.001
	No	n/N (%)	42/207 (20.3%)	165/207 (79.7%)			
History of kidney stone	Yes	n/N (%)	12/54 (22.2%)	42/54 (77.8%)	0.673	0.418-1.756	0.673
	No	n/N (%)	50/200 (25%)	150/200 (75%)			
Smoking	Yes	n/N (%)	10/40 (25%)	30/40 (75%)	1.038	0.476-2.267	0.925
	No	n/N (%)	52/214 (24.3%)	162/214 (75.7%)			
Painkiller intake	Yes	n/N (%)	20/49 (40.8%)	29/49 (59.2%)	2.677	2.677- 5.194	0.003
	No	n/N (%)	42/205 (20.5%)	163/205 (79.5%)			
Herbal medicine intake	Yes	n/N (%)	15/32 (46.8%)	17/32 (53.2%)	3.285	1.528-7.063	0.002
	No	n/N (%)	47/222 (21.2%)	175/222 (78.8%)			
Cancer History	Yes	n/N (%)	0/2 (0%)	2/2 (100%)	--	--	0.420
	No	n/N (%)	62/252 (24.6%)	190/252 (75.4%)			
Obesity	BMI<30	n/N (%)	54/201 (26.9%)	147/201 (73.1%)	0.484	0.214-1.092	0.076
	BMI≥30	n/N (%)	8/53 (15.1%)	45/53 (84.9%)			

The mean serum creatinine level of patients with CKD was 1.2 mg/dl as compared to non-CKD patients which was 0.73 mg/dl (reference range 0.6-1.1 mg/dl) (Table 3). The mean UACR of patients with CKD was 1007.85 mg/gm as compared to non-CKD patients which was 8.03 mg/gm (reference range 0-30 mg/gm). It shows that patients with CKD on average had higher creatinine levels. Similarly, patients who were found to have CKD had a mean age of 49.9 years as compared to non-CKD patients who had a mean age of 43.7. No significant difference was noted in the means of BMI between the two groups.

**Table 3:** Comparison of means across CKD sub-groups SD: standard deviation; UACR: urine albumin to creatinine ratio; BMI: body mass index; eGFR: estimated glomerular filtration rate; CKD: chronic kidney disease

Variable	CKD status	Mean ±SD	P-Value
Serum Creatinine	Yes	1.2035 ±0.74424	0.000
	No	0.7311 ±0.17510	
UACR mg/g	Yes	1007.85 ±2152.987	0.000
	No	8.03 ±7.818	
BMI	Yes	26.11 ±3.720	0.285
	No	26.81 ±4.684	
Age	Yes	49.95 ±14.916	0.002
	No	43.74 ±12.904	
eGFR ml/min	Yes	49 ±23	0.000
	No	85±14	

## Discussion

Despite being classified as a global epidemic by the World Health Organization (WHO) in 2008, chronic diseases remain a relatively neglected area of research in many developing nations. A recent study focusing on CKD revealed that over 800 million individuals worldwide are afflicted with CKD, with a significant imbalance in its prevalence between high-income and low-income countries [2]. In countries like Pakistan, where a comprehensive nationwide data collection system is lacking, obtaining reliable information about non-communicable diseases poses a considerable challenge. Our cross-sectional study, conducted on the urban population of Lahore, Pakistan, represents a crucial step forward. It aims to address this gap and provide insights into a region that significantly contributes to the global burden of disease.

The findings of this study, carried out on a cohort of 254 patients, offer valuable insights into the prevalence and determinants of CKD within the examined group. Notably, a considerable portion of the patients, totaling 24.4% or one in four, received a diagnosis of CKD. This prevalence underscores the necessity of comprehending the factors contributing to CKD and devising targeted interventions to combat this health issue. One noteworthy observation emerging from the study pertains to the gender distribution among the patients. While the number of male and female participants was nearly equivalent, with a slight majority of females (51.2%), the incidence of CKD did not exhibit significant bias toward either gender. This implies that CKD affects both males and females at a comparable rate within this particular population and highlights the importance of preventative measures for both genders. These findings are in contrast with numerous studies conducted in the Southeast Asian region which demonstrate a slightly higher prevalence of CKD among females [12].

A recent review of Pakistani literature carried out by Salman and Ashar showed that the overall prevalence of CKD across all age groups was 21.2% [8]. The highest reported prevalence was 29.9%, while the lowest was 12.5%. Among patients over 50 years of age, the prevalence was highest at 43.6%. Two studies [13,14] indicated a higher prevalence in males (62% and 54.4%, respectively), while two others [7,9] showed a higher prevalence in females (64% and 52%, respectively). Diabetic nephropathy was identified as the most common cause of CKD (27.1%), followed by CKD of unknown etiology (16.6%) and renal stone disease (12.4%). It was found that CKD is particularly prevalent among the elderly. The leading causes of CKD vary across studies due to differences in research centers, hospital settings, and urban locations. Common causes of CKD include diabetic nephropathy, CKD of unknown origin, and renal stone disease [8]. Our study did not find a significant association between a history of kidney stones and CKD. This can be attributed to the relatively small sample size as compared to other studies, an important limitation of our study.

Diabetes was an important high-risk factor for CKD in the study population, accounting for almost 43% (109/254) of the participants. Almost 36.6% (40/109) of diabetic patients were found to have evidence of CKD. A study by Imran et al. found the prevalence of CKD in urban Karachi, Pakistan to be 25.60% [9]. Their study also discovered a significant association between CKD and diabetes mellitus as well as hypertension (p=0.006). These findings underscore the importance of early detection and management of chronic kidney disease in urban populations. According to another study, CKD develops in nearly half of patients with type 2 diabetes mellitus and one-third with type 1 diabetes mellitus [15]. A similar study in India showed CKD prevalence to be 46% among diabetic patients [10].

Hypertension was one of the most common high-risk factors for CKD in our study population, accounting for almost 52% (131/254) of the participants. Global hypertension prevalence is a significant health concern, with estimates indicating that around 29% of the world's adult population had hypertension in 2000, and this number is projected to increase to 60% by 2025, totaling 1.56 billion individuals [16-18]. The burden of hypertension is expected to rise due to factors such as the aging population, increasing obesity rates, and decreased physical activity levels [18]. This increase is particularly notable in developing nations, where an 80% rise in prevalence is anticipated [19]. In our study, 30% (39/131) of hypertensive patients were found to have evidence of CKD. This prevalence of CKD in hypertensive patients is lower than in some other studies which indicates that among patients with hypertension, the prevalence of CKD ranges from 39.8% to 57.1% [20,21]. This difference may be attributed to the fact that hypertension in this study was defined as a blood pressure of 130/80 mmHg or higher, while some previous studies have used a threshold of over 140/90 mmHg.

A family history of CKD was present in 58.3% (149/254) of the studied population, of which 30.9% (n=46) were found to have CKD. CKD patients were 2.48 times more likely to have a positive family history of CKD as compared to patients who did not have CKD, and this was statistically significant (P<0.05). The prevalence of CKD in family members varies across studies. Studies indicate that the prevalence of CKD in first and second-degree relatives of hemodialysis patients ranges from 5.6% to 15.8% [22-24].

Available literature indicates a heightened risk of nephrotoxicity associated with the use of non-combination NSAIDs among patients with CKD. Additionally, NSAIDs can induce kidney injury through idiosyncratic reactions, such as acute interstitial nephritis. Specifically, pain relievers, particularly NSAIDs, have been found to be significantly linked to a 2.7-fold increased risk of kidney disease in the studied population. Many early and frequently referenced studies have documented the correlation between self-reported prior NSAID consumption by patients and the presence of CKD [25-28].

Several studies have shown that nephrotoxicity related to alternative medicines is on the rise globally, with cases of irreversible renal impairment reported in patients using such therapies [29]. In addition to the well-known risk factors mentioned previously, there is a notable association with the excessive use of herbal and hakim (herbal medicine practitioners) medications in this region. Statistical analysis of our study reveals that individuals using herbal medicine are 3.285 times more likely to develop CKD compared to non-users. In Pakistan, the unregulated nature of hakim medication often results in contamination with toxins, uncertain dosages, potential drug interactions, and misidentification of herbal species. The lack of safety testing obscures the full range of their adverse effects. For instance, there are over 600 flowering plants utilized as folk medication in this area [30]. Cultural and religious beliefs, myths, superstitions, the cost of allopathic medication, and limited access to equitable healthcare drive many individuals to seek traditional remedies. Addressing this issue requires a multifaceted approach, including the equitable distribution of medical resources, stringent regulation of phytomedicine, conducting safety trials for a better understanding of their effects, and training medical personnel to offer non-judgmental counseling to patients interested in alternative medicine.

Furthermore, a significant observation pertains to the distribution of BMI among the study participants. The majority of patients (79.1%) exhibited a BMI below 30. This challenges the prevailing notion that CKD is predominantly linked to obesity. Some studies have shown that obesity is a significant risk factor for CKD development, independent of factors like type 2 diabetes mellitus and hypertension [31]. Another systematic review and meta-analysis showed that obese individuals have a 1.81 times higher risk of CKD [32]. Instead, our study findings indicate that CKD can impact individuals across varying BMI categories, highlighting the complex nature of this condition. The relatively lower number of participants in the study with a BMI above 30 might explain the lack of a significant difference in CKD incidence among individuals with different BMIs.

One limitation of our research is its cross-sectional design, which lacked any follow-up procedures. However, patients diagnosed with CKD were referred to their preferred nephrologists for further evaluation and treatment. According to feedback from their nephrologists, all the patients under follow-up were confirmed to have CKD. Additionally, the study did not address the impact of non-GFR factors on creatinine levels, such as physical exertion, muscle mass, and dietary habits, potentially leading to inaccurate estimations of kidney function via eGFR [33]. Similarly, various factors and day-to-day variability can affect the albumin-to-creatinine ratio. The reference test, a timed urinary albumin excretion, is cumbersome and prone to collection errors, making it inconvenient and thus not performed. Furthermore, the age-related decline in eGFR was not considered, although our study population primarily consisted of younger individuals, with an average age of 45.26 ± 13.66 years.

## Conclusions

Our study revealed that CKD was prevalent in about one-fourth of the participants from the high-risk population of Lahore, indicating a high prevalence of the disease within society. This research provides valuable insights into the high prevalence of CKD and its associated factors among the population under investigation. The results emphasize the significance of inclusive screening initiatives aimed at individuals with hypertension, diabetes, and a family history of CKD. It is essential to address these risk factors through targeted interventions to reduce the impact of CKD in the community. Furthermore, discouraging the unintended use of NSAIDs, herbal remedies, and alternative treatments is crucial, as there is an elevated risk of kidney disease in this population.
